# Ofatumumab-mediated CD20^+^ B-cell depletion promotes neuromuscular junction functional recovery and sustains long-term efficacy in refractory generalized myasthenia gravis

**DOI:** 10.3389/fphar.2025.1728721

**Published:** 2025-11-27

**Authors:** Zhuangzhuang Ren, Yuping Chen, Yudan Liu, Yan Wang, Shengjie Xu, Yanhua Yang, Feng Qiu

**Affiliations:** Senior Department of Neurology, Chinese PLA Hospital, Beijing, China

**Keywords:** generalized myasthenia gravis, ofatumumab, B-cell depletion, neuromuscular junction transmission, functional recovery, long-term efficacy

## Abstract

**Objective:**

This study aimed to evaluate the long-term efficacy and safety of the fully human anti-CD20 monoclonal antibody ofatumumab in refractory generalized myasthenia gravis, with focus on its potential to promote recovery of neuromuscular junction transmission function.

**Methods:**

Fourteen refractory patients treated at the Eighth Medical Center of the Chinese PLA General Hospital from March 2023 to June 2024 were included. All had relapsed after maintaining Minimal Manifestation Status for over 1 year and showed inadequate response to immunosuppressants. Ofatumumab 20 mg was administered at weeks 0, 1, 2, and 4, followed by additional doses every 4–6 months according to symptoms and CD19-positive B-cell reconstitution. Low-frequency 3-Hz repetitive nerve stimulation of the facial, accessory, and axillary nerves was conducted at baseline and month 12. MG-ADL, QMG, MGC scores and B-cell and T-cell levels were monitored.

**Results:**

The compound muscle action potential (CMAP) decrement of the predominantly involved nerve and the mean decrement across the three tested nerves improved at month 12, indicating enhanced neuromuscular junction transmission. MG-ADL, QMG, and MGC scores declined from month 1, with earlier improvement in bulbar, limb, and neck muscle groups. All patients discontinued corticosteroids by month 12 while remaining clinically stable. B cells stayed at low levels, whereas T-cell counts showed no significant change. Treatment was well tolerated.

**Conclusion:**

Ofatumumab provides durable clinical benefit and steroid-sparing effects, and its sustained depletion of CD20-positive B cells may facilitate recovery of neuromuscular junction transmission. These findings support its potential application within the field of regenerative pharmacology.

## 1 Introduction

Myasthenia gravis (MG) is an antibody-mediated, T-cell–dependent autoimmune disease involving multiple complement pathways. Immature B cells are activated by antigens present on the postsynaptic membrane of the neuromuscular junction (NMJ), and with the help of helper T cells, they differentiate into memory B cells and antibody-secreting plasma cells. The acetylcholine receptor (AChR) antibody and muscle-specific receptor tyrosine kinase (MuSK) antibody have clear pathogenic roles. These antibodies cause NMJ dysfunction, clinically presenting as fluctuating muscle weakness in the ocular and/or limb muscles (Yi et al., 2018; Dresser et al., 2021). Although the majority of patients experience significant symptom improvement with conventional immunotherapies such as corticosteroids and non-steroidal immunosuppressants, 10%–20% of refractory MG patients show limited response to conventional immunotherapies. These patients may not tolerate the drugs, experience adverse reactions, or have recurring symptoms. Adjusting the medication dosage often fails to provide sufficient symptom relief, making it difficult to achieve the treatment goals of symptom remission or Minimal Manifestation Status (MMS) (Tran et al., 2021). Currently, the available treatment options are limited, and there is a need for more effective therapeutic solutions.

Most B cells express CD19 and/or CD20 on their surface, and peripheral blood CD19^+^ B cell levels are commonly used in clinical practice as a surrogate pharmacodynamic marker for CD20^+^ B-cell depletion (Forsthuber et al., 2018). Monoclonal antibodies such as rituximab and ofatumumab exert immune-modulating effects by targeting CD20 to treat MG by reducing the production of pathogenic antibodies and thereby helping restore NMJ function (Piehl et al., 2022). Rituximab, a monoclonal antibody targeting CD20 on B cells, effectively reduces the risk of MG relapse and delays the progression of neurological dysfunction. However, it requires intravenous infusion, and infusion-related adverse reactions are common, which increases the risk of infections, thus limiting its clinical use (Levy and Mealy, 2021). Ofatumumab, the latest fully human anti-CD20 monoclonal antibody, has lower immunogenicity compared to rituximab and offers more convenient administration. It is administered subcutaneously, without direct transport through the bloodstream to the spleen, preserving B lymphocytes in the marginal zone of the spleen, which helps maintain the body’s ability to fight infections. Ofatumumab has been widely used in the treatment of multiple sclerosis with good safety profiles (Samjoo et al., 2022; Kramer et al., 2023). Additionally, several case reports have explored the use of ofatumumab to treat other neuroimmune diseases, demonstrating its promising therapeutic potential (Podesta et al., 2024; Zhang et al., 2023; Gou et al., 2023).

Based on this, we applied ofatumumab to 14 patients with refractory generalized myasthenia gravis (gMG), systematically evaluating its long-term efficacy and safety, assessing changes in NMJ transmission using low-frequency RNS, and analyzing recovery patterns across different muscle groups. The aim was to provide more comprehensive evidence for evaluating treatment efficacy in refractory gMG and to explore the potential role of B-cell-targeted immunotherapy in promoting functional recovery of the NMJ.

## 2 Study subjects and methods

### 2.1 Study subjects

This study enrolled 14 patients with refractory generalized myasthenia gravis who were treated at the Department of Neurology, Eighth Medical Center of the Chinese PLA General Hospital from March 2023 to June 2024. All patients had previously achieved Minimal Manifestation Status (MMS) for ≥1 year following treatment but subsequently experienced relapse, and showed poor responses to adjustments in immunosuppressive therapies, including tacrolimus, cyclosporine, and cyclophosphamide. The study was approved by the Ethics Committee of the Eighth Medical Center of the Chinese PLA General Hospital (S-2023-008-01), conducted in full accordance with the Declaration of Helsinki, and all patients provided written informed consent.

#### 2.1.1 Inclusion criteria

Patients were required to meet the diagnostic criteria of the Chinese Guidelines for the Diagnosis and Treatment of Myasthenia Gravis (2020 edition) (Chang, 2021). In addition, inclusion required fulfillment of the 2016 MGFA International Consensus definition of refractory myasthenia gravis (Sanders et al., 2016), in which patients show no improvement or even worsening despite adequate dose and duration of corticosteroids plus at least two other immunosuppressive agents, and continue to experience clinically significant symptoms or treatment-limiting adverse effects.

#### 2.1.2 Exclusion criteria

Presence of other autoimmune or neurological diseases that may interfere with MG assessment; severe dysfunction of major organs such as the heart, liver, or kidneys, or psychiatric disorders; active infection or uncontrolled severe chronic infection; recent vaccination history; presence of malignancy; inability to complete follow-up or clinical assessments.

### 2.2 Methods

#### 2.2.1 Data collection

Data were collected from the patients’ electronic medical records. Clinical data included the age of onset, gender, patient characteristics, follow-up duration, and the most recent symptoms at the time of the final examination. Laboratory data included antibody types, CD19^+^ B-cell percentages, and total T-cell percentages. Treatment information included the therapeutic regimen, dates, number of ofatumumab courses, dosages, and any adverse reactions. Follow-up data included MG-ADL, Quantitative Myasthenia Gravis score (QMG), Myasthenia Gravis Composite (MGC) scores, and corticosteroid usage, as well as RNS findings and the dosage of prednisone acetate; RNS follow-up data were missing in three patients due to intolerance of the examination, whereas all other clinical data were complete.

#### 2.2.2 Ofatumumab treatment protocol

Ofatumumab 20 mg was administered by subcutaneous injection at weeks 0, 1, 2, and 4. Subsequent injections were administered every 4–6 months when the MG-ADL score had increased by more than 3 points and the CD19^+^ B-cell percentage exceeded 2%.

#### 2.2.3 Scale evaluations

Two experienced senior neurologists assessed patients using the MG-ADL, QMG, and MGC scales before treatment and at 1, 3, 6, and 12 months post-treatment. Additionally, individual scores for bulbar, limb, neck, respiratory, and ocular muscles were evaluated using the QMG scale. Each follow-up evaluation window was ±3 days. The MG-ADL scale assesses quality of life, while the QMG and MGC scales assess disease severity. The MG-ADL scale consists of 8 items, scored on a 4-point scale (0–3), with a total score ranging from 0 to 24. A higher score indicates a worse quality of life. The QMG scale includes 13 items, scored on a 4-point scale (0–3), with a total score ranging from 0 to 39. A higher score indicates greater severity. The MGC scale consists of 10 items, scored using a weighted 4-point scale (0–9), with a total score ranging from 0 to 50. A higher score indicates greater severity (Barohn et al., 1998; Burns et al., 2010; Wolfe et al., 1999).

#### 2.2.4 Immune cell testing method

Testing was conducted according to the manufacturer’s instructions. A total of 20 μL of antibody mixture containing PerCP/Cyanine5.5 anti-human CD3 (BioLegend, United States) and APC anti-human CD19 (BioLegend, United States) was added into an absolute counting tube, followed by 50 μL of EDTA-anticoagulated whole blood using a reverse pipetting technique. After mixing, the samples were incubated for 15 min at room temperature in the dark, then 450 μL of lysing solution (BioLegend, United States) was added. The samples were mixed again and incubated for another 15 min at room temperature in the dark. Flow cytometric acquisition and analysis were performed on a BD FACSCanto flow cytometer using BD FACSCanto software. BD™ Cytometer Setup and Tracking (CST) beads were used for daily instrument calibration and quality control, ensuring a coefficient of variation <5%.

#### 2.2.5 Repetitive nerve stimulation for assessing NMJ functional transmission

According to standard neurophysiological procedures, 3-Hz low-frequency RNS was performed on the facial, accessory, and axillary nerves to evaluate neuromuscular junction transmission. Examinations were scheduled at baseline and at 12 months after treatment (±10 days). To reduce the impact of inter-individual differences in nerve involvement patterns, the nerve with the greatest baseline compound muscle action potential (CMAP) decrement was defined as the predominant involved nerve and used as the primary endpoint for analysis. In addition, the mean CMAP amplitude decrement across the facial, axillary, and accessory nerves was calculated as an index of overall NMJ transmission recovery, representing bulbar, proximal limb, and neck–shoulder muscle groups, respectively.

#### 2.2.6 Statistical analysis methods

All statistical analyses and data visualizations were performed using R (RStudio v4.4.2). The normality of continuous variables and of paired differences was assessed using the Shapiro–Wilk test. Normally distributed continuous variables are presented as mean ± standard deviation and were compared using paired t-tests or independent-samples t-tests according to the data structure. Non-normally distributed continuous variables are expressed as median (interquartile range); paired comparisons were conducted using the Wilcoxon signed-rank test, and between-group comparisons were conducted using the Mann–Whitney U test. For repeated-measures clinical indicators with three or more time points, overall differences were assessed using the Friedman test, followed by Bonferroni-corrected Wilcoxon signed-rank tests for *post hoc* pairwise comparisons. For indicators with only two time points, paired t-tests or Wilcoxon signed-rank tests were applied based on the normality of paired differences. All tests were two-sided, and p < 0.05 was considered statistically significant.

## 3 Results

### 3.1 Clinical characteristics of patients with refractory gMG

This study included 14 patients with refractory gMG, consisting of 6 males and 8 females, with an average age of 54.0 ± 16.7 years and a disease duration of 7.0 (3.0, 12.5) years. Among them, five patients were MuSK-positive, including three males and two females, with a mean age of 62.8 ± 14.2 years and a disease duration of 11.2 ± 9.2 years. Nine patients were AChR-positive, including three males and six females, with a mean age of 49.7 ± 16.1 years and a disease duration of 7.3 ± 4.6 years. There were no significant differences in age, gender, disease duration, or disease severity between the MuSK antibody-positive and AChR antibody-positive groups. The median pre-treatment prednisone acetate dosage for all 14 patients was 50.0 mg (Table 1).

**TABLE 1:** Clinical characteristics of 14 refractory gMG patients.

Characteristics	Total patients	MuSK-Ab(+) patients	AChR-Ab(+) patients	P-value
	(n = 14)	(n = 5)	(n = 9)	
Gender				0.343^a^
Male	6 (43%)	3 (60%)	3 (34%)	
Female	8 (57%)	2 (40%)	6 (66%)	
Age	54.0 ± 16.7	62.8 ± 14.2	49.7 ± 16.1	0.148^b^
Disease duration	7.0 (3.0, 12.5)	11.2 ± 9.2	7.3 ± 4.6	0.308^b^
Prednisone	50.0 (40.5, 51.25)	50.0 ± 9.4	50.0 (40.0, 50.0)	0.421^b^

a Fisher’s Exact Test; b Independent samples t-test; c Mann–Whitney U test.

### 3.2 Changes in peripheral CD19^+^ B cells and total T cells

**FIGURE 1:**
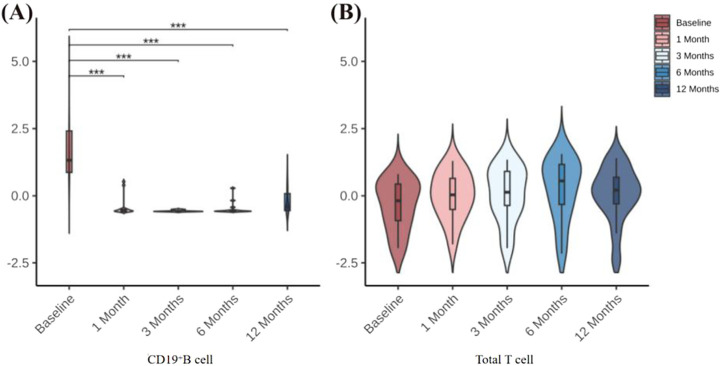
**(A)** Changes in CD19^+^ B-cell percentages before treatment and at 1, 3, 6, and 12 months after treatment. **(B)** Changes in total T-cell percentages before treatment and at 1, 3, 6, and 12 months after treatment. *P < 0.05; **P < 0.01; **P < 0.001.

We found by flow cytometry that, in all 14 patients with refractory gMG, the percentage of peripheral blood CD19^+^ B cells began to show a sustained decrease starting at 1 month after ofatumumab treatment. The percentages at 1, 3, 6, and 12 months post-treatment were all significantly lower than those before treatment. There were no significant changes in the percentage of total T cells before and after treatment (Figure 1).

### 3.3 Neuromuscular junction functional recovery assessed by low-frequency RNS

**FIGURE 2:**
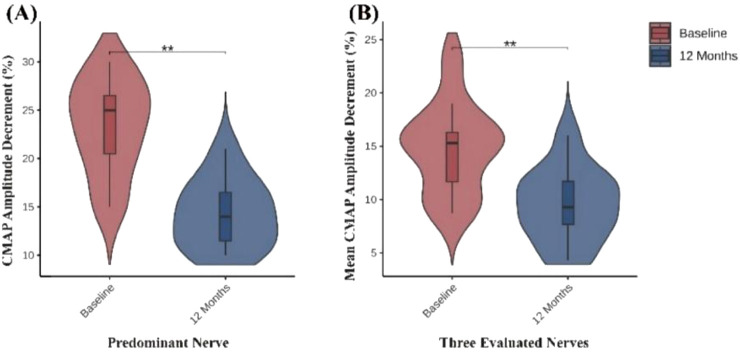
Changes in neuromuscular transmission assessed by low-frequency RNS. **(A)** CMAP amplitude decrement (%) of the predominant nerve significantly decreased at 12 months compared with baseline. **(B)** Mean CMAP amplitude decrement (%) of the three evaluated nerves (facial, axillary, and accessory nerves) also showed a significant reduction at 12 months. *P < 0.05; **P < 0.01; ***P < 0.001.

Among the 14 patients included in this study, 11 completed low-frequency RNS examinations before treatment and at 12-month follow-up. The remaining 3 patients were unable to complete follow-up testing due to intolerance to stimulation-related discomfort. To accurately reflect the overall changes in NMJ transmission, we analyzed the CMAP amplitude decrement of the predominantly affected nerve for each patient during low-frequency RNS, and additionally calculated the mean CMAP amplitude decrement across the facial, axillary, and accessory nerves to minimize potential bias from evaluating a single nerve. Compared with baseline, all 11 patients who completed follow-up demonstrated varying degrees of recovery of NMJ transmission function at 12 months. The CMAP decrement of the predominantly affected nerve showed a significant reduction, and the mean decrement across the three nerves exhibited a consistent downward trend, indicating recovery of NMJ transmission function (Table 2; Figure 2).

**TABLE 2:** Changes in CMAP amplitude decrement at baseline and 12 months in 11 patients undergoing low-frequency RNS.

Variable	Baseline (%)	12 months (%)	p-value
	(n = 11)	(n = 11)	
Predominant nerve decrement	25.0 (20.0, 27.0)	14.0 (11.0, 17.0)	0.003
Mean three-nerve decrement	15.3 (11.3, 16.3)	9.3 (7.3, 11.7)	0.003

P-values from Wilcoxon signed-rank tests comparing baseline vs. 12 months.

### 3.4 Clinical symptom improvement and long-term maintenance of efficacy

**FIGURE 3:**
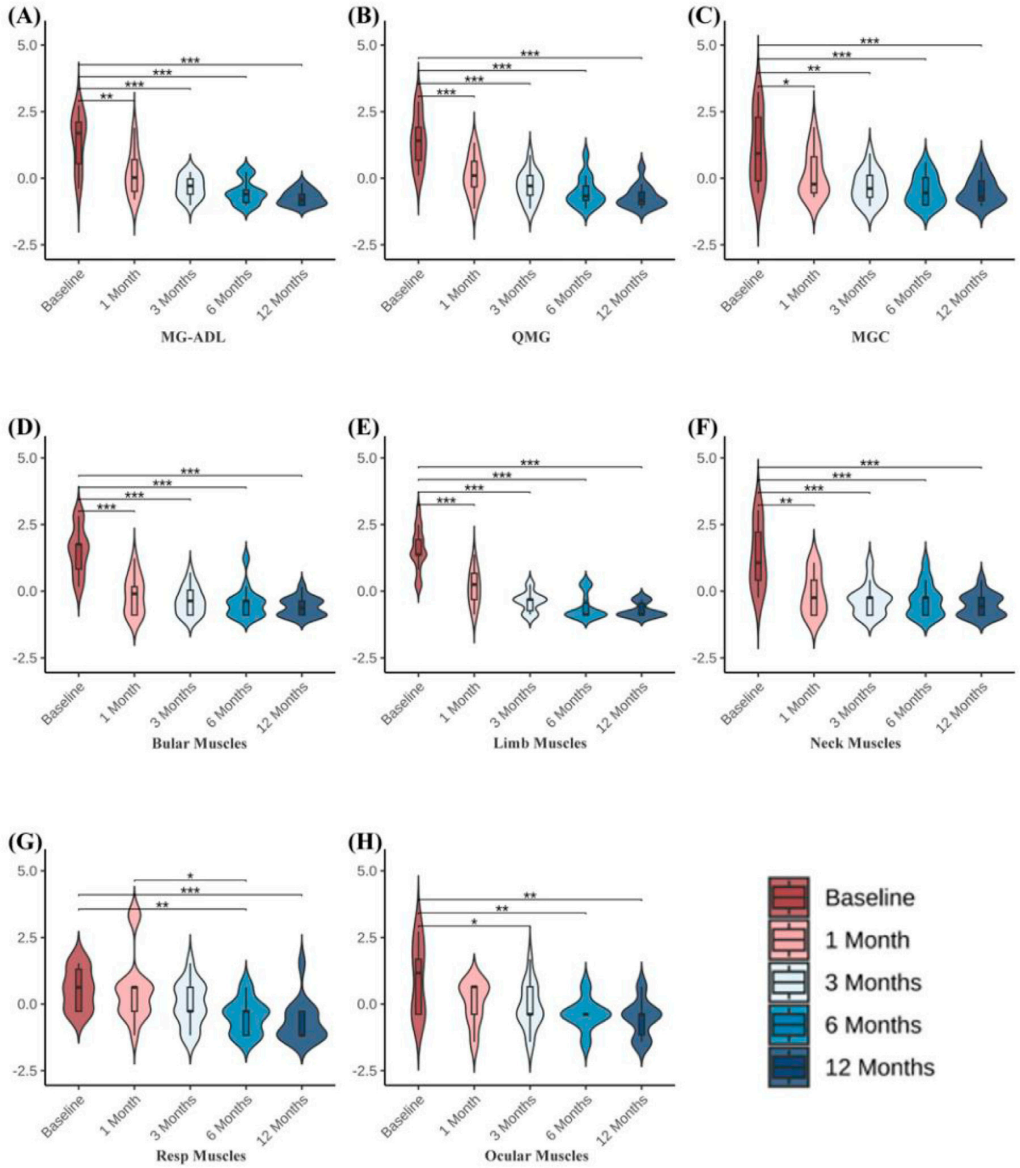
**(A–C)** MG-ADL, QMG, and MGC scores before treatment and at 1, 3, 6, and 12 months following ofatumumab treatment in 14 gMG patients. **(D–H)** QMG scale individual muscle scores for bulbar muscles, limb muscles, neck muscles, respiratory muscles, and ocular muscles before treatment and at 1, 3, 6, and 12 months after ofatumumab treatment in 14 gMG patients. *P < 0.05; **P < 0.01; ***P < 0.001.

**FIGURE 4:**
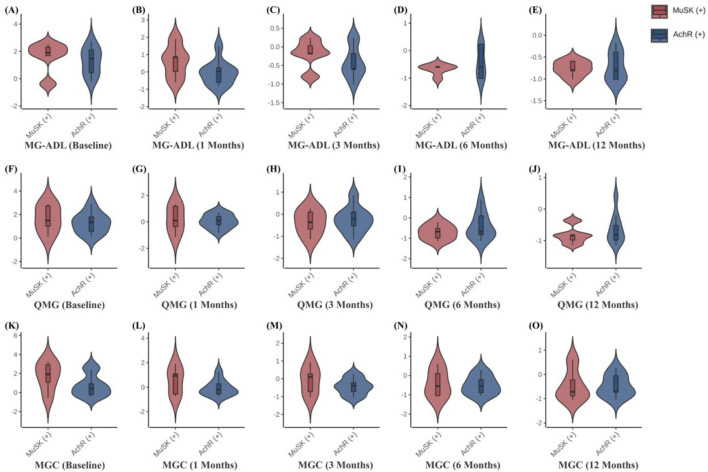
**(A–O)** Comparison of MG-ADL, QMG, and MGC scores at different time points in 5 MuSK antibody-positive patients and 9 AChR antibody-positive patients.

Clinical scale assessments revealed that ofatumumab treatment resulted in significant symptom improvement in refractory gMG patients at each follow-up visit. The MG-ADL, QMG, and MGC scores at 1, 3, 6, and 12 months post-treatment were significantly lower than those before treatment. Furthermore, improvements in bulbar, limb, and neck muscles occurred more rapidly than in respiratory and ocular muscles. The scores for bulbar, limb, and neck muscles were significantly lower at 1, 3, 6, and 12 months post-treatment compared to pre-treatment. The respiratory muscle scores at 6 and 12 months post-treatment were significantly lower than pre-treatment, with the 6-month score being significantly lower than the 1-month score. The ocular muscle scores at 3, 6, and 12 months post-treatment were significantly lower than pre-treatment (Table 3; Figure 3). Ofatumumab treatment led to consistent symptom improvement in both MuSK antibody-positive gMG patients and AChR antibody-positive gMG patients. There were no significant differences in the MG-ADL, QMG, MGC scores, or individual muscle scores for bulbar, limb, neck, respiratory, and ocular muscles between the MuSK antibody-positive and AChR antibody-positive groups at baseline, 1, 3, 6, and 12 months post-treatment (Table 4; Figure 4).

**TABLE 3:** Changes in MG-ADL, QMG, MGC scores, and individual muscle scores for bulbar muscles, limb muscles, neck muscles, respiratory muscles, and ocular muscles in 14 gMG patients before treatment and at 1, 3, 6, and 12 months after ofatumumab treatment.

Variable	Baseline	1 Month	3 Months	6 Months	12 Months	P-value
	n = 14	n = 14	n = 14	n = 14	n = 14	
MG-ADL	13.00 (6.75, 15.25)	5.00 (2.00, 9.00)	3.50 (1.75, 5.25)	2.00 (0.00, 3.75)	1.00 (0.00, 2.25)	**<0.001** ^**a**^
QMG	16.50 (10.75, 21.25)	8.00 (4.75, 12.00)	5.50 (2.75, 8.00)	3.00 (1.75, 6.50)	2.00 (1.00, 4.25)	**<0.001** ^**a**^
MGC	12.00 (4.75, 21.50)	5.00 (3.00, 12.25)	4.00 (1.50, 7.25)	3.00 (0.00, 7.00)	2.00 (1.00, 6.00)	**<0.001** ^**a**^
Bulbar muscles	5.00 (3.00, 5.50)	1.50 (0.00, 2.25)	1.00 (0.00, 2.00)	1.00 (0.00, 1.00)	0.50 (0.00, 1.00)	**<0.001** ^**a**^
Limb muscles	4.00 (3.75, 5.25)	2.00 (1.00, 3.00)	1.00 (0.00, 1.00)	0.00 (0.00, 1.25)	0.00 (0.00, 1.00)	**<0.001** ^**a**^
Neck muscles	3.00 (2.00, 5.25)	1.00 (0.00, 2.25)	1.00 (0.00, 1.00)	1.00 (0.00, 1.25)	0.50 (0.00, 1.00)	**<0.001** ^**a**^
Respiratory muscles	2.00 (1.00, 3.00)	2.00 (1.00, 2.00)	1.00 (0.75, 2.00)	1.00 (0.00, 1.00)	0.00 (0.00, 1.00)	**<0.001** ^**a**^
Ocular muscles	2.50 (1.00, 3.25)	2.00 (1.00, 2.00)	1.00 (1.00, 2.00)	1.00 (1.00, 1.00)	1.00 (0.00, 1.00)	**0.007** ^**a**^
CD19^+^ B cells	12.11 (8.46, 20.09)	0.16 (0.03, 0.58)	0.00 (0.00, 0.31)	0.10 (0.03, 0.47)	1.15 (0.09, 5.09)	**<0.001** ^**a**^
Total T cells	69.47 ± 13.60	74.64 ± 12.80	75.18 ± 15.11	78.10 ± 16.81	75.32 ± 15.16	0.640^b^

MG-ADL, myasthenia gravis activities of daily living; QMG, quantitative myasthenia gravis score; MGC, Myasthenia Gravis Composite. a: Friedman test; b: repeated-measures ANOVA. All tests are two-sided with α = 0.05; p < 0.001 is reported as “<0.001”. Asterisks indicate comparisons versus baseline (Bonferroni-corrected). Statistically significant results (p < 0.05) are shown in bold.

**TABLE 4:** Comparison of MG-ADL, QMG, and MGC scores at different time points before treatment and at 1, 3, 6, and 12 months after ofatumumab treatment in 5 MuSK antibody-positive patients and 9 AChR antibody-positive patients.

	Variable	MuSK-Ab (+)	AChR-Ab (+)	P-value
		(n = 5)	(n = 9)	
Baseline	MG-ADL	12.60 ± 5.59	11.00 ± 4.74	0.580^a^
	QMG	18.00 ± 7.58	15.67 ± 5.63	0.523^a^
	MGC	16.80 ± 9.26	10.89 ± 7.01	0.201^a^
1 Month	MG-ADL	7.80 ± 4.55	4.67 ± 3.39	0.167^a^
	QMG	8.80 ± 6.76	7.56 ± 3.43	0.651^a^
	MGC	9.60 ± 6.88	5.89 ± 3.82	0.212^a^
3 Months	MG-ADL	4.00 ± 1.87	2.89 ± 2.09	0.343^a^
	QMG	5.00 ± 3.67	5.67 ± 3.81	0.756^a^
	MGC	5.80 ± 4.82	3.67 ± 2.78	0.308^a^
6 Months	MG-ADL	2.00 (2.00, 2.00)	2.00 (0.00, 6.00)	0.525^b^
	QMG	2.80 ± 2.39	4.67 ± 4.15	0.379^a^
	MGC	4.00 ± 4.42	3.33 ± 2.92	0.738^a^
12 Months	MG-ADL	1.20 ± 0.84	1.44 ± 1.59	0.757^a^
	QMG	2.00 ± 1.87	3.33 ± 3.12	0.405^a^
	MGC	3.60 ± 4.04	3.44 ± 2.70	0.932^a^

MuSK, Muscle-specific receptor tyrosine kinase; AChR, acetylcholine receptor; MG-ADL, myasthenia gravis activities of daily living; QMG, quantitative myasthenia gravis score; MGC, Myasthenia Gravis Composite. a: independent-samples t-test; b: Mann–Whitney U test. All tests are two-sided with α = 0.05; *p* < 0.001 is reported as “<0.001.” Statistically significant results (*p* < 0.05) are shown in bold.

### 3.5 Significant reduction in corticosteroid (prednisone acetate) dosage

Ofatumumab treatment significantly reduced corticosteroid usage in refractory gMG patients. By the third month, the average prednisone acetate dose for the 14 patients had decreased by 59%. By the sixth month, the average dose had decreased by 84%, and by the 12th month, all patients had stopped corticosteroid use, while maintaining stable clinical status. This result not only indicates that ofatumumab effectively improves symptoms but also that it reduces patients’ reliance on corticosteroids, thus lowering the risk of potential side effects associated with long-term corticosteroid use.

### 3.6 Safety evaluation of ofatumumab

In terms of safety, only one of the 14 patients experienced mild adverse symptoms, specifically nausea, which resolved spontaneously. The other patients tolerated the treatment well and did not experience any adverse reactions.

## 4 Discussion

Refractory gMG is characterized by recurrent and fluctuating disease progression, with some patients unable to achieve effective control despite adjustments in immunosuppressive therapy. This population faces a significant disease burden (Waters et al., 2019). This study systematically evaluated the long-term efficacy and safety of ofatumumab in patients with refractory generalized myasthenia gravis, as well as its impact on NMJ transmission. Notably, alongside clinical improvement, patients exhibited a marked reduction in low-frequency repetitive nerve stimulation (RNS) decrement at 12 months, indicating recovery of NMJ transmission efficiency. This electrophysiological evidence provides key support for the role of B-cell–targeted therapy in restoring NMJ function, helps elucidate the mechanistic action of ofatumumab in neuromuscular disorders, and complements the sustained clinical benefits observed in this study.

The therapeutic efficacy of ofatumumab has been demonstrated in several previous studies (Li et al., 2023). Rituximab, a widely used CD20^+^ B-cell–depleting agent, is traditionally administered at a standard regimen of 375 mg/m^2^ weekly for four consecutive weeks, and subsequent research has explored various alternative dosing cycles (Robeson et al., 2017; Anderson et al., 2016; Díaz-Manera et al., 2012). Multiple studies have suggested that, when clinically indicated, retreatment should be considered approximately 6 months after completion of the initial treatment cycle (Tandan et al., 2017). Based on this rationale, in our study, the 14 patients with refractory gMG received subcutaneous ofatumumab 20 mg at weeks 0, 1, 2, and 4, with an additional dose administered every 4–6 months depending on clinical symptoms and changes in CD19^+^ B-cell percentages. We observed that within 1 year of treatment, patients achieved both optimal clinical improvement and sustained B-cell depletion. In addition, no significant changes were detected in the percentage of total T cells in peripheral blood. Previous studies have shown that B-cell–targeted depletion can modulate T-cell subset–mediated immune responses. For example, the immunomodulatory effects of rituximab on T cells in MG patients have been repeatedly reported (Jing et al., 2019; Damato et al., 2023). Furthermore, both rituximab and ofatumumab have demonstrated regulatory effects on T-cell populations in other autoimmune diseases (von Essen et al., 2022; Yahyazadeh et al., 2022; Bounia and Liossis, 2021). Alterations in T-lymphocyte subsets in MG patients have also been associated with disease outcomes (Liu et al., 2025). In our study, the lack of significant change in total T-cell percentages may indicate that ofatumumab exerts relatively mild effects on T-cell subsets in refractory gMG, or that T-cell proportions gradually stabilize as disease activity improves. Because this study primarily focused on CD20^+^ B-cell depletion, detailed analysis of T-cell subsets was not further pursued.

B-cell depletion therapy in MG is thought to act primarily by reducing the production of AChR and MuSK antibodies, thereby attenuating complement-mediated postsynaptic membrane injury, and may also facilitate recovery of NMJ transmission by modulating the immune microenvironment and local inflammatory responses at multiple levels (Chamberlain et al., 2021; San and Jacob, 2023; Cavalcante et al., 2024). In this study, we selected the nerve with the greatest baseline decrement as the individualized predominant nerve for primary evaluation, in order to minimize bias arising from differences in the distribution of neuromuscular involvement among patients, as this nerve best represents the main pathological site in each individual. In addition, to reflect the overall NMJ functional status, we calculated the mean decrement of the facial, accessory, and axillary nerves—three nerves with relatively high positivity rates—as a supplementary analysis (Witoonpanich et al., 2006), which further strengthened the robustness of the results. Although only 11 patients completed the full RNS follow-up due to factors such as discomfort during stimulation, both analytic approaches showed a consistent trend of improvement, supporting the reliability of the electrophysiological findings. While this study did not perform statistical correlation analyses between electrophysiological measures and clinical outcomes, the simultaneous appearance of electrophysiological and clinical improvement during follow-up suggests that sustained immune modulation may provide the basis for the gradual restoration of NMJ function.

It is important to highlight that there have been reports indicating ofatumumab’s efficacy in AChR antibody-positive MG patients. Additionally, rituximab has shown particular benefits for MuSK antibody-positive MG patients, while demonstrating partial efficacy in AChR antibody-positive MG patients (Ma et al., 2024). However, the efficacy of ofatumumab in refractory gMG patients with either MuSK or AChR antibody positivity, as well as its impact on different muscle group functions, remains unclear. This study provides comprehensive, long-term data on the efficacy of ofatumumab in refractory gMG patients with different antibody profiles and muscle function recovery. We found that, in the absence of significant baseline differences between AChR antibody-positive and MuSK antibody-positive patients, ofatumumab treatment yielded similar clinical benefits in both groups, with no significant differences between them.

Both AChR and MuSK antibodies are produced by B cells with the assistance of T cells and other lymphocytes. Self-activated B cells play an important role in the pathogenesis of MG, so B cell-targeted therapies can be effective (Dalakas, 2008). AChR antibodies exert their effects through three mechanisms: complement activation, antigen modulation, and functional blocking of ACh-AChR binding. Complement activation is an effector function of immunoglobulins, which can lead to the formation of the membrane attack complex, subsequently damaging the NMJ membrane. MuSK, a transmembrane protein located on the postsynaptic membrane of the NMJ, plays a critical role in AChR aggregation. Its major subtype, IgG4, differs significantly in molecular characteristics from the AChR antibody subtype, IgG1, in pathological mechanisms (Ma et al., 2024). Ofatumumab is a fully humanized IgG1 monoclonal antibody targeting CD20^+^ B cells. Compared to rituximab, ofatumumab binds to two distinct, non-continuous epitopes on CD20, with slower dissociation from the CD20 antigen, resulting in stronger complement-dependent cytotoxicity, providing effective and sustained effector activity (Klein et al., 2014; Semple et al., 2019). This may explain why ofatumumab shows significant efficacy in both AChR antibody-positive and MuSK antibody-positive patients. Further, larger-sample prospective randomized controlled studies are needed to confirm this potential mechanism.

The effect of ofatumumab on different muscles in gMG has not been reported. The main symptoms of MG are related to the bulbar muscles, limb muscles, neck muscles, respiratory muscles, and ocular muscles. In our study, we analyzed the scores for these five domains based on the QMG scale. The bulbar muscle score significantly improved at 1 month post-treatment, the limb muscle score improved at 3 months, and the neck muscle score improved at 3 months. We noted that the improvement in the respiratory and ocular muscle scores was slower, showing improvement at 6 months post-treatment. This may be due to the refractory nature of MG cases and the fact that respiratory muscles are more prone to involvement (Kaminski et al., 2004). Therefore, in terms of muscle group improvement, ofatumumab had a more rapid effect on bulbar, limb, and neck muscles, suggesting that ofatumumab may have superior therapeutic value for gMG patients with predominant bulbar, limb, and neck muscle symptoms. Furthermore, MG crisis-related pre-crisis status is characterized by exacerbation of bulbar or respiratory muscle symptoms within 2 weeks and the potential risk of progression to myasthenic crisis (MC). These patients typically require inclusion in a standardized treatment system for timely monitoring and intervention. Cholinesterase inhibitors must be adjusted for symptomatic treatment, and rapid-acting therapeutic strategies should be employed to quickly improve symptoms and prevent MC progression. Our study found that ofatumumab showed good efficacy in bulbar muscles, and its use during pre-crisis status may have potential value in preventing progression to the MC stage (Stetefeld and Schroeter, 2019).

Pharmacokinetic analysis shows that ofatumumab 20 mg, administered subcutaneously with repeated dosing, has a steady-state half-life of approximately 16 days (Awada et al., 2024). Although an initial weekly dosing regimen may place an economic burden on some patients, we found that the QMG, MG-ADL, and MGC scores significantly decreased at 1 month, 3 months, 6 months, and 12 months post-treatment. With additional doses every 4–6 months, symptoms remained well-controlled within 1 year, and corticosteroid use gradually decreased. By 12 months, all patients had discontinued corticosteroid use, further validating the rationality of the current treatment regimen and the benefits for patients. The patients in this cohort are still being followed up, with some having received ofatumumab treatment for up to 18 months, and their symptoms remain stable.

Taken together, the RNS findings suggest that ofatumumab may not only improve clinical symptoms and reduce corticosteroid requirements in the long-term management of refractory gMG, but also contribute to the recovery of NMJ transmission, providing deeper electrophysiological support for its clinical benefits. However, this study has limitations, including the relatively small sample size and the inability of some patients to complete RNS follow-up due to discomfort during electrical stimulation. Future studies with larger sample sizes, multicenter participation, and longer follow-up periods, as well as basic research integrating immune remodeling, complement regulation, and postsynaptic membrane stability, are needed to further clarify the long-term role of B-cell–targeted therapy in NMJ structural and functional restoration, thereby providing more precise and individualized treatment options for MG patients.

## 5 Conclusion

This study shows that, after ofatumumab treatment, low-frequency decrement on RNS was significantly reduced, reflecting recovery of NMJ transmission function. Ofatumumab can continuously improve clinical symptoms in refractory gMG patients and achieves faster and more significant efficacy in the bulbar, limb, and neck muscles. Moreover, this treatment can effectively reduce corticosteroid usage, lowering the risk of long-term steroid-related adverse effects. Both AChR antibody-positive and MuSK antibody-positive patients can achieve the same clinical benefits, with good safety and tolerability. In summary, ofatumumab not only provides stable clinical improvement in refractory gMG but is also accompanied by recovery of NMJ transmission, offering new evidence to support long-term management in these patients.

## Data Availability

The original contributions presented in the study are included in the article/supplementary material, further inquiries can be directed to the corresponding author.
